# Announcing the new launch of *The Egyptian Journal of Neurology, Psychiatry and Neurosurgery*

**DOI:** 10.1186/s41983-018-0002-6

**Published:** 2018-04-25

**Authors:** M. Ossama Abdulghani, Ahmed M. Abdelalim, Khaled O. Abdulghani

**Affiliations:** Helwan University School of Medicine, Cairo, Egypt

We are honored to announce the new launch of the *Egyptian Journal of Neurology, Psychiatry and Neurosurgery* (EJNPN), the official publication of the Egyptian Society of Neurology, Psychiatry and Neurosurgery (ESNPN), a peer-reviewed, open access journal publishing articles in the field of clinical neurosciences, including neurology, psychiatry and neurosurgery. The journal presents cutting edge research on clinical, technical, ethical and social aspects of clinical neurosciences.

First, we must acknowledge our colleagues who are joining us in the editorial office currently in Cairo, Egypt. They represent the true motivators and dynamos for the journal. Leading the scientific publication in the neurosciences field in Egypt, the Middle East and North Africa; the EJNPN was first published in 1960. Throughout the years, the journal has been indexed by several indexing services, including EMBASE and Scimago journal ranking. At the meantime, we are working closely with other relevant indexing services including PubMed Central and Web of Science to ensure that articles published in the EJNPN will be available in their databases when appropriate.

Taking the next step towards better indexing, ranking, reader’s exposure and targeting more authors from around the world, the journal moved to Springer Open – Springer Nature, through an agreement with the Egyptian Knowledge Bank as an open access and free-of-charge journal. Open access allows the journal to reach a larger set of authors and readers (Suber 2005) with higher downloads and citations, leading to a higher Impact Factor (Hitchcock n.d.; Brody and Harnad n.d.). This also helps avoiding any influence of the economy on the reader’s ability to access articles (Tan-Torres Edejer 2000).

Authors hold copyright for their work and grant anyone the right to reproduce and disseminate the article, provided that it is correctly cited. This complies with the policies of several funding bodies including the Wellcome Trust, NIH and Howard Hughes Medical Institute (Janka5 2018; Janka6 2018; Janka7 2018; Janka8 2018).

The EJNPN operates a double-blind peer-review system, where the reviewers do not know the names or affiliations of the authors and the reviewer reports provided to the authors are anonymous. Submitted manuscripts will generally be reviewed by two to three experts who will be asked to evaluate whether the manuscript is scientifically and ethically sound and coherent, whether it duplicates already published work, and whether or not the manuscript is sufficiently clear for publication. Reviewers will also be asked to indicate how interesting and significant the research is. The Editors will reach a decision based on these reports, and where necessary, they will consult with members of the Editorial Board (Janka9 2018). Articles will be published online soon upon acceptance and hopefully soon, after being listed, in PubMed Central/PubMed.

We welcome your research and scientific work to be submitted to the EJNPN (Janka10 2018). We promise you visibility, flexibility and speedy publication, together with better promotion and press coverage.

M. Ossama Abdulghani—The Editor-in-Chief, EJNPN.

Ahmed M. Abdelalim and Khaled O. Abdulghani—The Associate Editors, EJNPN.
